# Clinical Researches on the Exhibition of Kermes Mineral in Pulmonary Affections

**Published:** 1846-03

**Authors:** 


*Clinical Researches on the exhibition of Kermes Mineral in Pulmonary Affections.*—Dr. Herpin has not found kermes so efficient in diseases of the pulmonary structure as it is stated to be; but in affections of the superior air passages he has derived from it the greatest benefit. He goes so far as to consider kermes mineral as a specific against the disorders of the respiratory tubes. In true croup Dr. H. has employed it as the "only" means of treatment, and obtained the most satisfactory results from its administration. In idiopathic and chronic laryngitis, kermes has again proved most servicable. Dr. H. has also used the medicine with benefit in some cases of deafness, caused by chronic obstruction of the Eustachian tube; the dose at which the drug has been exhibited in these several cases, varied between one and twelve grains in the twenty-four hours; on the average from three to six grains daily. When three grains are taken in one dose, sicknesss is generally the immediate result. The drug must be given in extremely small doses to avoid nausea, and to obtain merely alterative effects; the hour after meals is the most favorable to its exhibition.—*Med. Times in Med. News.*

